# Mapping of novel chromosomal regions associated with atopy

**DOI:** 10.1186/1710-1492-10-S1-A52

**Published:** 2014-03-03

**Authors:** Cynthia Kanagaratham, John Ren, Pierre Camateros, Rafael Marino, Rob Sladek, Silvia Vidal, Danuta Radzioch

**Affiliations:** 1 Department of Human Genetics, McGill University, Montreal Quebec, Canada; 2 Department of Microbiology and Immunology, McGill University, Montreal, Quebec, Canada; 3 Faculty of Medicine, Division of Experimental medicine, McGill University, Montreal, Quebec, Canada

## Background

**Figure 1:**
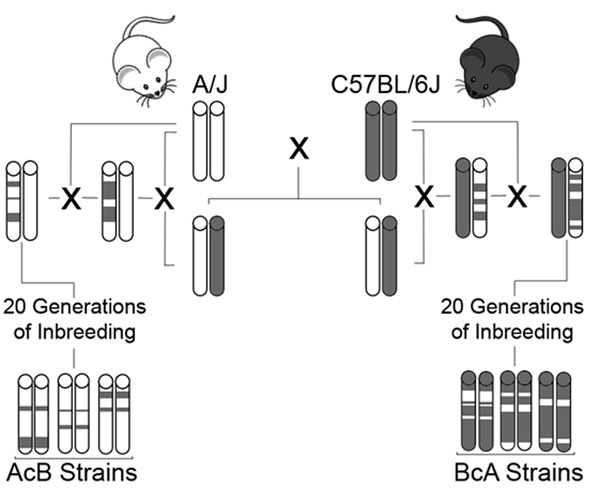
Generation of 33 RCS from atopic A/J and nonatopic C57BL/6J strains (adapted from [2])

A panel of recombinant congenic strains (RCS) of mice can be used to study an array of disease related phenotypes [1]. We have used a panel of 33 AcB/BcA RCS, derived from parental strains A/J and C57BL/6J (Figure 1), to study phenotypes of allergic asthma that are difficult to segregate in the human population, such as airway hyperresponsiveness [2]. Each recombinant strain is fully inbred and contains approximately 12.5% of the genome from one parental strain on the background of the other parental strain. Here we present our findings for mapping chromosomal regions associated with atopy, another phenotype of allergic asthma.

## Methods

Naïve mice from each RCS were phenotyped for atopy by measuring plasma IgE concentration by ELISA. RCS mice were genotyped at 1215 markers that spanned the entire genome. Using the log transformed phenotype values and genotyping data, a marker-by-marker association analysis was performed to identify associations between the strain phenotype and genotype, while correcting for major background strain. Within the phenotype associated loci, candidate genes were selected based on the presence of coding mutations between the sequences of the two parental strains.

## Results

**Figure 2:**
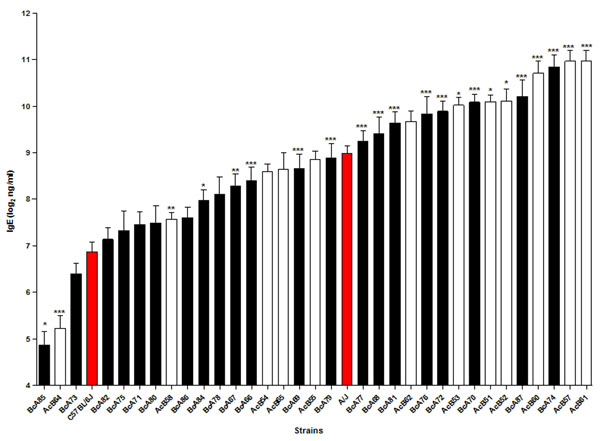
Strain distribution pattern of parental strains A/J and C57BL/6J (red bars), 12 AcB strains (white bars), and 21 BcA strains (black bars). Significance was calculated by one-way ANOVA by comparing each RCS to its major genetic donor parental strain. *, ** and *** represents p<0.05, p<0.01 and p<0.001, respectively, post Bonferroni correction.

A/J and C57BL/6J strains have significantly different plasma IgE concentrations. A/J mice have higher plasma IgE levels, making them a good model of atopic individuals. Among the 33 RCS, a wide distribution in plasma IgE concentrations was observed (Figure 2). Genotype-phenotype analysis identified one region on chromosome 3 as significantly associated with atopy. This region contains a total of six protein coding genes of which four have coding variants in their sequences between A/J and C57BL/6J strains.

## Conclusions

To the best of our knowledge, we have identified a novel candidate loci associated with atopy. Future plans of our study include functionally validating the importance of our candidate genes, candidate locus, and of chromosome 3 in atopy. Our results demonstrate that using a genetically unique panel of RCS we can identify candidate genes that are in common and unique to the various phenotypes of allergic asthmatics.

## References

[B1] Fortin A Diez E Rochefort D Laroche L Malo D Rouleau GA Recombinant congenic strains derived from A/J and C57BL/6J: a tool for genetic dissection of complex traits Genomics 2001 10 21 35 11374899 10.1006/geno.2001.6528

[B2] Camateros P Marino R Fortin A Martin JG Skamene E Sladek R Identification of novel chromosomal regions associated with airway hyperresponsiveness in recombinant congenic strains of mice Mamm Genome 2010 10 28 38 20012967 10.1007/s00335-009-9236-z

